# Construction and Verification of Nomogram Model for Lung Adenocarcinoma With ≤ 5 Bone-Only Metastases Basing on Hematology Markers

**DOI:** 10.3389/fonc.2022.858634

**Published:** 2022-06-01

**Authors:** Chunliu Meng, Fang Wang, Minghong Chen, Hongyun Shi, Lujun Zhao, Ping Wang

**Affiliations:** ^1^ Department of Radiation Oncology, Tianjin Medical University Cancer Institute and Hospital, National Clinical Research Center for Cancer, Tianjin Key Laboratory of Cancer Prevention and Therapy, Tianjin’s Clinical Research Center for Cancer, Tianjin, China; ^2^ Department of Radiation Oncology, Affiliated Hospital of Hebei University, Baoding, China; ^3^ Department of Radiation Oncology, The Rich Hospital Affiliated of Nantong University, Nantong, China

**Keywords:** lung adenocarcinoma, bone-only metastasis, prognostic factors, nomogram model, prediction

## Abstract

**Objectives:**

This retrospective study investigated prognostic factors in advanced lung adenocarcinoma (LUAD) with one to five bone-only metastasis (BOM) and developed a nomogram model to estimate patient survival.

**Methods:**

We investigated patients with advanced LUAD with one to five bone-only metastasis at the initial diagnosis and diagnosed between 2013 and 2019 in two hospitals. A formula named Risk-H was constructed using hematological variables screened by LASSO-Cox regression analysis in the internal set and verified by the external set. Two nomogram models were developed by clinical variables selected by LASSO-Cox regression analysis with or without Risk-H in the internal set. The concordance index (C-index), calibration curves, time-dependent receiver operating characteristic (ROC) analysis, area under the curve (AUC), and decision curve analysis (DCA) were formulated to verify nomogram models. The primary endpoint was overall survival.

**Results:**

We finally included 125 and 69 patients, respectively, in the internal and external sets for analysis. The following were significant hematology prognostic factors and were included in the Risk-H formula: alkaline phosphatase and albumin, leukocyte. Four clinical factors, including loss of weight, sensitive mutation status, T and N stage, with or without Risk-H were used to establish nomogram models. C-index, calibration curves, ROC analysis, AUC, and DCA showed the addition of hematological data improved the predictive accuracy of survival.

**Conclusions:**

Pretreatment peripheral blood indexes may be a meaningful serum biomarker for prognosis in LUAD. The addition of Risk-H to the nomogram model could serve as a more economical, powerful, and practical method to predict survival for LUAD patients with one to five BOM.

## Introduction

Lung adenocarcinoma (LUAD) is a common malignant tumor, and accounts for 40%-50% of lung cancer cases worldwide (1). The rate of distant metastasis is high, and a common site of metastasis is the skeletal system (2, 3).

In LUAD patients with skeletal metastasis, many factors may impinge on the quality of life and performance status, leading to the duration of survival varying greatly. These include epidemiological history, distribution of metastasis, molecular alteration, histopathological type, and the number of metastases, hematological markers, and so on (4–7). Notably, the prognosis is also affected by the metastatic spread of LUAD to sites other than bone, such as the brain or liver (8). Thus, paying close attention to patients with bone-only metastasis (BOM) is best for studies of survival time in LUAD with skeletal metastasis, although this has rarely been considered.

Considering advanced LUAD, oncologists and radiologists are more likely to focus on patients with oligometastatic disease characterized by reduced metastatic potential with a limited number of metastatic sites (9), which renders it amenable to local treatment (LT). Several clinical trials and multiple retrospectives series have reported favorable outcomes of LT in highly selected oligometastatic non-small cell lung cancer (NSCLC) patients (10–18). So, the National Comprehensive Cancer Network guidelines recommend LT as standard and homogeneous treatment strategy for them. However, fewer attempts have been made to investigate whether clinical variables could contribute to the selection for more superior prognosis of patients.

In the present study, we investigated the hematology data, demographic, and clinical information of LUAD patients with ≤ 5 BOM from two hospitals, recorded at the initial diagnosis. Additionally, to guide physicians in estimating the survival time of these patients, a nomogram model basing on a comprehensive hematological formula was developed.

## Materials & Methods

### Selection of Study Population

Data were retrospectively collected from the records of consecutive patients who received a diagnosis of advanced NSCLC in two hospitals from 2013 to 2019. Clinical staging of the disease was conducted renewedly with reference to the eighth edition for tumor-node-metastasis (TNM) classification (19), at the time of data collection. The inclusion criteria in this study were: (1) a diagnosis of LUAD confirmed from pathological or cytological specimens, or both; (2) evidences of bone metastasis confirmed by imaging examinations, such as plain radiograph, CT, PET-CT, MRI, and bone scan, or a bone biopsy performed during surgery; (3) the number of bone metastases was ≤ 5; (4) a data of gene mutation status identified *via* next-generation sequencing; (5) did not receive immunotherapy in the first-line. Patients were excluded if they had second primary tumor; a site of metastasis other than bone; without gene sequence result; or incomplete medical records.

### Definition of Special Concept

In this study, positively sensitive mutations (SM^+^) included: epidermal growth factor receptor (*EGFR*) exon 19 deletion, *EGFR* exon 21 Leu858Arg mutation, and anaplastic lymphoma kinase (*ALK*) mutation. *EGFR* uncommon mutations, such as exon 18 mutations, exon 20 insertion mutations and so on, no-targeted therapy mutations or without any mutation, were defined as sensitive mutations negative (SM^–^).

### Hematology Markers

Laboratory examinations including routine blood test data, hepatic, and renal function test data of patients were collected before initial treatment. The calculation formulas of neutrophils to lymphocyte ratio (NLR), platelet to lymphocyte ratio (PLR), and systemic inflammatory index (SII) were as follows: NLR = neutrophil number (10^9^/L)/lymphocyte count (10^9^/L); PLR = number of platelets (10^9^/L)/number of lymphocytes (10^9^/L); SII = number of platelets(10^9^/L) × number of neutrophils (10^9^/L)/number of lymphocytes (10^9^/L). Corrected calcemia was computed according to the formula: c-Ca = measured Ca + (40-albumin)/40. The best cutoff values for albumin, alkaline phosphatase (ALP), leukocyte, PLR, NLR, SII, and c-Ca were obtained according to overall survival (OS).

### First-Line Systemic Treatment Strategy

All patients with *EGFR* non-sensitive mutations, no-targeted therapy mutations, or without mutation underwent first-line chemotherapy after confirmation of the initial LUAD diagnosis. The treatment included platinum-based doublet chemotherapy such as pemetrexed or paclitaxel combined with cisplatin, carboplatin, or nedaplatin. Each chemotherapy session was separated by an interval of 3 to 4 weeks.

Patients with *EGFR*-sensitive mutations (exon 19 deletion, exon 21 Leu858Arg mutations) were administered first-line treatment with *EGFR* tyrosine kinase inhibitors (TKIs), such as gefitinib, erlotinib, and icotinib; or with chemotherapy mentioned above and then TKIs after disease progression. All patients with *ALK* mutation were administered first-line treatment with crizotinib, or with chemotherapy as aforesaid and then TKI after disease progression.

### Data Analysis and Statistical Considerations

OS was the primary endpoint, defined as the time from the date of diagnosis until death or the last follow-up. The follow-up schedule began from the time of treatment to the final follow-up on November 22, 2021. The data on the date of death or at the final follow-up visit were acquired from hospital records or through direct correspondence with the family of patients. R 4.1.1 software and SPSS 24.0 software were used to perform the statistical analyses. The chi-squared test (or Fisher’s exact test as applicable) and independent-samples T test were used to compare the clinical characteristics between the internal and external groups. OS was estimated using Kaplan-Meier method and between-group difference in OS was assessed using log-rank test. The optimal cutoff values of hematology markers were determined using the package “survminer” based on OS. LASSO-Cox regression analysis was performed to select the optimal prognostic factors using packages “glmnet,” “survival,” and “MASS” and the backward-forward stepwise method. The “predict” function of package “survival” was used to calculate the risk-score of each patient.

Nomograms, including clinical variables alone or clinical variables plus Risk-H, were constructed by using the package “regplot”. The concordance index (C-index) and calibration curves were evaluated to assess the consistency between the predicted and observed probabilities using package “pec”. The time-dependent receiver operating characteristic (ROC) analysis and area under the curves (AUC) were evaluated to assess the discrimination using packages “survivalROC” and “riskRegression”. The decision curve analysis (DCA) was formulated to evaluate the clinical practicality of constructed models using package “ggDCA”. All P-values were two-sided, with P < 0.05 considered statistically significant.

## Results

### Patient Characteristics

**Figure 1:**
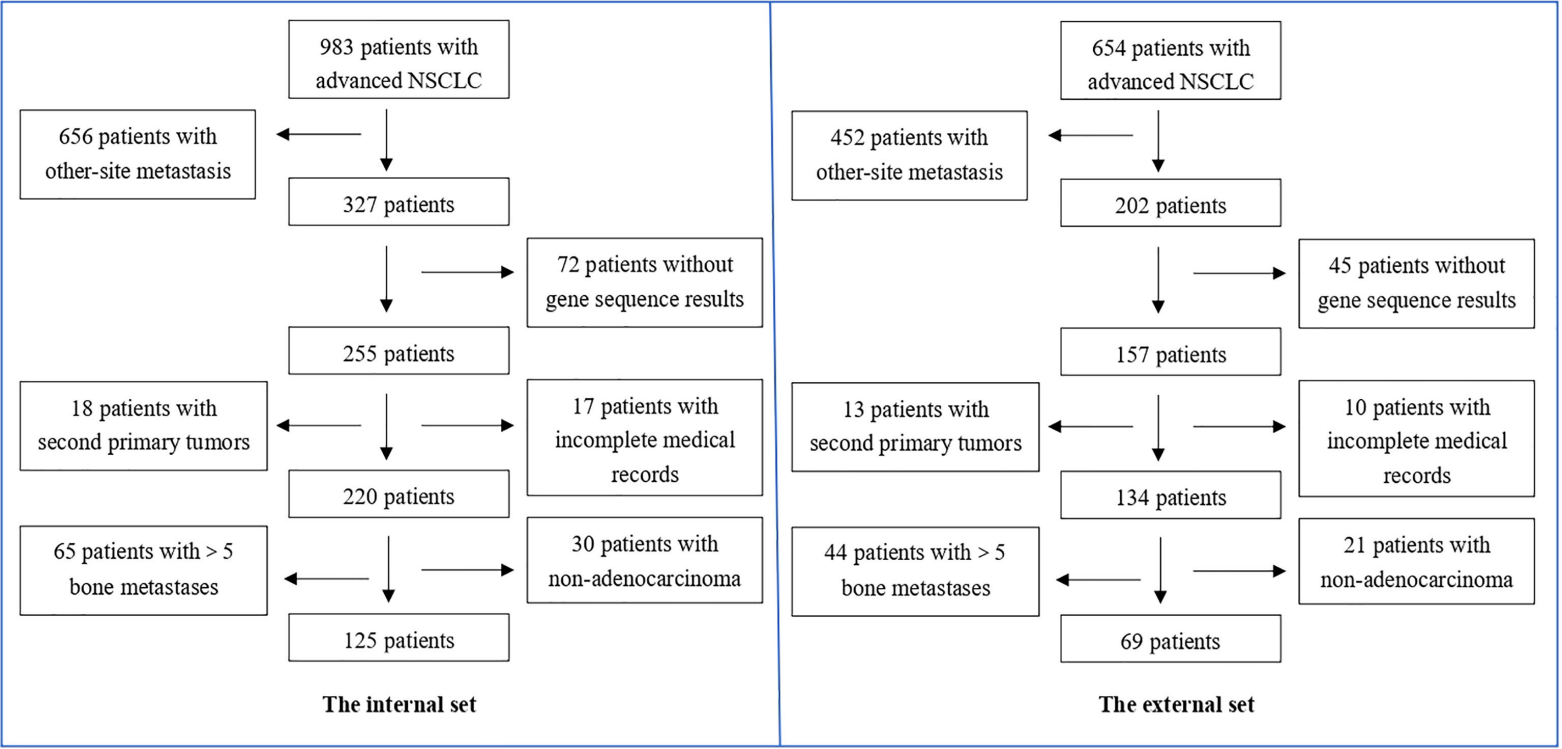
Patient selecting process for the internal set and the external set.

Data were collected for 983 patients and 654 patients with advanced NSCLC, who had been treated in two hospitals from January 2013 to December 2019. The detailed patients selecting process is shown in **Figure 1**. Eventually, 125 and 69 patients, respectively, were enrolled in the internal set and the external set. The demographic and clinic-pathological features of patients are displayed in **Table 1**. The median follow-up time, median OS, and 1-, 3-, 5-year survival rates were 35.5 vs. 43.0 months, 28.3 vs. 32.7 months, 87% vs. 88.4%, 43.4% vs. 49.0%, and 17.4% vs. 13.0%, respectively, in the internal set and external set.

**Table 1:** Clinical characteristics of patients.

Characteristics	The internal set (n=125) No. of patients (%)	The external set (n=69) No. of patients (%)	*P*-value
Gender (female/male)	55/70 (44.0/56.0)	32/37(46.4/53.6)	0.750
Age (<65/≥65)	92/33 (73.6/26.4)	40/29 (58.0/42.0)	0.025*
Mean ± SD	58.1 ± 9.60	62.1 ± 8.83	0.005*
KPS (<80/≥80)	11/114 (8.8/91.2)	11/58 (15.9/84.1)	0.133
Smoking history	46 (36.8)	32 (46.4)	0.193
N stage			0.360
N0-1	39 (31.2)	26 (37.7)	
N2-3	86 (68.8)	43 (62.3)	
T stage			0.748
T1-2	86 (68.8)	54 (78.3)	
T3-4	39 (31.2)	15 (21.7)	
Number of bone metastasis			0.543
1	49 (39.2)	24 (34.8)	
2-5	76 (60.8)	45 (65.2)	
Gene alternation status			0.051
* EGFR*-sensitive mutations	64 (51.2)	25 (36.2)	
* ALK* mutation	8 (6.4)	1 (1.5)	
* EGFR* unsensitive mutations	10 (8.0)	6 (8.7)	
Other mutations	7 (5.6)	4 (5.8)	
No	36 (28.8)	33 (47.8)	
Antiresorptive drugs	104 (83.2)	48 (69.6)	0.027*
ALP (U/L)	117.5 ± 55.55	95.4 ± 35.81	0.001*
Albumin (g/L)	41.9 ± 4.73	40.2 ± 4.72	0.017*
c-Ca	2.3 ± 0.14	2.3 ± 0.10	0.340
Leukocyte (10^9^/L)	7.5 ± 2.50	7.1 ± 2.29	0.294
NLR	3.5 ± 3.03	3.7 ± 2.39	0.614
PLR	185.1 ± 89.53	204.5 ± 129.39	0.271
SII	966.0 ± 714.59	1050.3 ± 943.18	0.485

*P < 0.05; KPS, karnofsky performance status scores; EGFR, epidermal growth factor receptor; ALK, anaplastic lymphoma kinase; PLR, platelet to lymphocyte ratio; NLR, neutrophils to lymphocyte ratio; SII, systemic inflammatory index; ALP, alkaline phosphatase.

### Risk-H Construction for OS

In the internal set, the optimal cutoff values of hematology markers were determined based on OS and the result showed that high albumin (p=0.020) was a favorable indicator of OS, whereas high ALP (p=0.005), high c-Ca (p=0.015), and high leukocyte (p=0.039) were accompanied by inferior OS. But the NLR (p=0.113), PLR (p=0.219), and SII (p=0.113) level had no significant statistical effects for survival (**Table 2**).

**Table 2:** Cutoff value and univariate Cox analysis of hematology markers in the internal set.

Characteristics	Cutoff	Categories	*P*-value
ALP	88.00	High (≥ 88.00) vs. Low (< 88.00)	0.005*
Albumin	42.80	High (≥ 42.80) vs. Low (< 42.80)	0.020*
c-Ca	2.39	High (≥ 2.39) vs. Low (< 2.39)	0.015*
Leukocyte	5.31	High (≥ 5.31) vs. Low (< 5.31)	0.039*
NLR	2.34	High (≥ 2.34) vs. Low (< 2.34)	0.113
PLR	249.69	High (≥249.69) vs. Low (<249.69)	0.219
SII	398.35	High (≥ 398.35) vs. Low (< 398.35)	0.113

*P < 0.05; PLR, platelet to lymphocyte ratio; NLR, neutrophils to lymphocyte ratio; SII, systemic inflammatory index; ALP, alkaline phosphatase.

**Figure 2:**
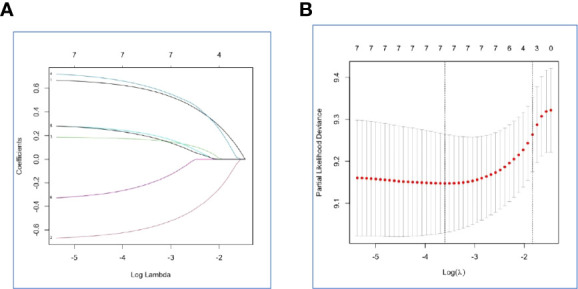
Construction of the Risk-H by the least absolute shrinkage and selection operator (LASSO) model in the internal set. **(A)** The LASSO-Cox regression model was used to generate the prognostic scoring system named Risk-H. **(B)** Ten-fold cross-validation for tuning parameter selection in the LASSO model *via* minimum criteria and 1-SE criteria.

**Figure 3:**
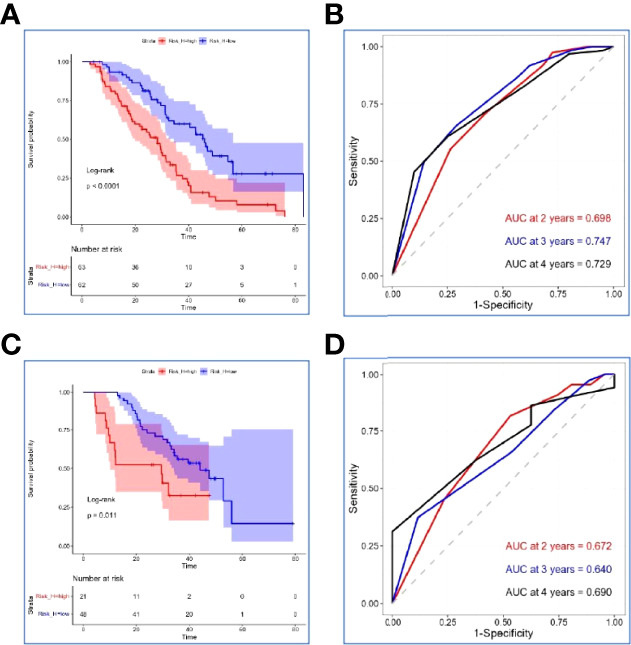
Verification of the Risk-H in the internal set and external set. **(A, C)** Kaplan–Meier survival analyses of Risk-H in the internal set and the external set. **(B, D)** Risk-H performance in time-dependent receiver operating characteristic (ROC) analysis in the internal set and the external set.

NLR, PLR, and SII had been reported as important prognostic factors for OS, so, these three variables and four other variables with p value < 0.05 were all included in the LASSO-Cox regression model to select the optimal prognostic variables in the internal set. Finally, ALP, albumin, and leukocyte were significantly independent prognostic factors, and were included to formulate the risk scoring system (**Figures 2A, B**, AIC value=666.31, p=8.176×e-06). A formula named Risk-H was constructed as follows: Risk-H=1 * HR-value (ALP) * HR-value (Albumin) * HR-value (Leukocyte) (**Table 3**). According to the median value of risk score (2.783129), patients in the internal set were divided into low-risk and high-risk groups and the median OS were 46.0 and 28.3 months, respectively (p<0.001, **Figure 3A**). Patients in the external set were calculated risk score according to the Risk-H formula and **Table 3** and were divided into low- and high-risk groups based on the median score mentioned above. This significant prognostic difference was also observed (p=0.011, **Figure 3C**). The prognostic accuracy of Risk-H was evaluated using time-dependent ROC analysis, yielding comparable AUC values between the internal and external sets with 2-, 3-, and 4-year AUC values of 0.698 vs. 0.672, 0.747 vs. 0.640, 0.729 vs. 0.690, respectively (**Figures 3B, D**), which confirmed the excellent prognostic power of Risk-H in another heterogeneous population.

**Table 3:** Factors included in the Risk-H formula.

Characteristics	Level	Coefficient	HR-value	*P*-value
ALP	1=low	0.7154	1	0.00521*
	2=high		2.0450	
Albumin	1=low	- 0.7557	1	0.00121*
	2=high		0.4697	
Leukocyte	1=low	1.0236	1	0.01724*
	2=high		2.7831	

*P < 0.05; ALP, alkaline phosphatase. Risk-H = 1*HR-value (ALP) *HR-value (Albumin) *HR-value (Leukocyte).

### Selecting of the Optimal Factors for Prognosis

**Figure 4:**
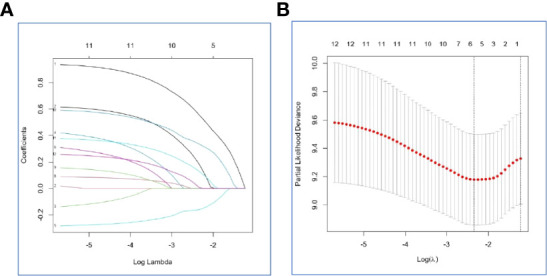
Selection of the optimal prognostic factors for survival in the internal set by the LASSO model. **(A)** The LASSO-Cox regression model was used to select the prognostic factors. **(B)** Ten-fold cross-validation for tuning parameter selection in the LASSO model *via* minimum criteria and 1-SE criteria.

In the internal set, Risk-H, and clinical variables, such as gender, age, smoking, karnofsky performance status scores, loss of weight, gene mutation status, T stage, N stage, number of bone metastases, antiresorptive drugs treatment, and weight bearing bone metastasis were included in the LASSO-Cox regression model to select optimal prognostic factors associated with OS and PFS using the backward-forward stepwise method (**Figures 4A, B**). After adjusting clinical characteristics, Risk-H (p < 0.001) remained as an independent negative prognostic indicator for OS. Beside Risk-H, another four clinical variables, including loss of weight, SM status, T stage, and N stage were added to establish the optimum model with the smallest AIC value (660.98, p=1.117×e-06) (**Table 4**). However, the significant effect of Risk-H on progression free survival (PFS) was not observed in the univariate analysis in the internal set (p=0.051).

**Table 4:** Factors included in the nomogram models.

Characteristics	Coefficient	HR-value	*P*-value
Risk-H (high vs. low)	1.0202	2.7738	1.6×e-05*
Loss of weight (yes vs. no)	0.6201	1.8591	0.0359*
SM status (- vs. +)	0.5402	1.7163	0.0211*
T stage (T3-4 vs. T1-2)	0.3701	1.4479	0.1280
N stage (N2-3 vs. N0-1)	0.3535	1.4240	0.1432

*P < 0.05; SM, sensitive mutations.

### Development and Validation of Nomogram Models

**Figure 5:**
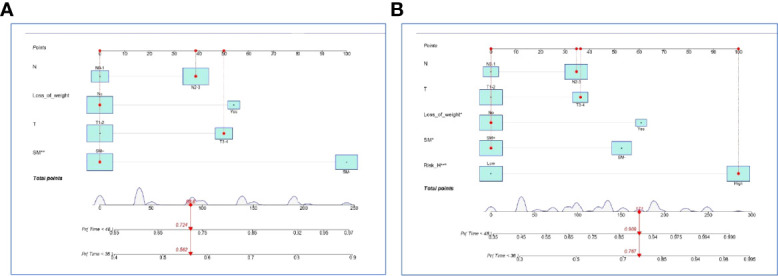
Nomogram models established by clinical variables alone **(A)** and plus Risk-H **(B)** in the internal set.

**Figure 6:**
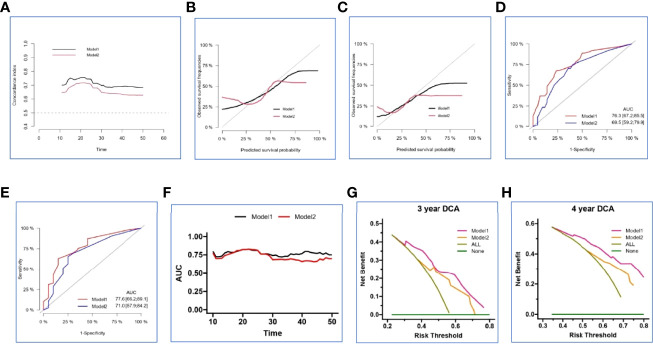
Validation and comparison of two nomogram models in the internal set. The time-dependent concordance index (C-index) **(A)**, 3- and 4-year calibration curves **(B, C)**, 3- and 4-year ROC analysis **(D, E)**, time-dependent area under the curve (AUC) **(F)**, 3- and 4-year decision curve analysis (DCA) **(G, H)** basing on Model 1 and Model 2.

In the internal set, nomogram models were established to predict the survival probability of LUAD with ≤ 5 BOM using four clinical variables mentioned above with or without Risk-H (Model 1 vs. Model 2). **Figures 5A, B** demonstrated an example of using the two nomogram models to predict the survival probability of a given patient which revealed that the addition of Risk-H to nomogram harbored improved predictive accuracy for survival when compared with that of clinical factors alone. The consistency between the predicted and observed probabilities were also improved as demonstrated by time-dependent C-index (**Figure 6A**) and 3- and 4-year calibration curves (**Figures 6B, C**).

We generated ROC analysis to assess the discrimination abilities of two nomograms and the result displayed nomogram constructed by clinical variables plus Risk-H had higher AUC value in predicting 3- and 4-year survival probability in the internal set (**Figures 6D, E**). Furthermore, time-dependent AUC curve also proved the superiority of combined prediction model again (**Figure 6F**).

Because the calibration curves and ROC analysis are based on the sensitivity and specificity of the predictive model, they cannot recognize false positive and false negative cases. While DCA was widely adopted to assess the clinical utility and net clinical benefits when the predictive model guides clinical practice. So, we performed 3- and 4-year DCA to evaluate the clinical utility and net clinical benefits that two nomograms would bring to patients and the results revealed that the integrated nomogram was significantly superior to the clinical nomogram in the internal set (**Figures 6G, H**). To sum up, the above results elucidated that the integrated nomogram had better predictive ability for the survival probability of LUAD patients with 1-5 BOM.

## Discussion

In the current study, we established a nomogram model using hematological and clinical data to predict the life expectancy of advanced LUAD patients with one to five BOM. A total of 125 and 69 cases, respectively, were included in the internal set and the external set. The following were significant hematology prognostic factors and were included in the Risk-H formula: alkaline phosphatase and albumin, leukocyte. Four clinical factors, including loss of weight, sensitive mutation status, T and N stage, with or without Risk-H were used to establish nomogram models in the internal set. C-index, calibration curves, ROC analysis, AUC, and DCA showed the addition of hematological data improved the predictive accuracy of survival.

Several hematological markers were reported to suggest a poor prognosis for lung cancer after bone metastasis including hypoalbuminemia, increased ALP and tumor-markers, and systemic inflammation, as evidenced by hyperleucemia, neutrophilia or high C-reactive protein (CRP) level (6, 7, 20–23). In the present study, a formula named Risk-H was established using peripheral blood data, and the high risk significantly reduced the survival time.

In year 2018, one prospective clinical trial investigated the bone, muscle, and metabolic parameters in patients with synchronous bone metastasis from lung cancer (7). The decrease of HbA1c, increase of DKK1 and serum calcium were poor prognostic factors for OS independently of common predictors. In the recent study which explored the prognosis factors of NSCLC with BOM, hypoalbuminemia significantly influenced patients’ survival (6). In our study, bone metabolic index ALP and nutritional index albumin were included in the Risk-H formula. Hence, these results indicated that supported treatment, such as anti-bone metabolism and nutritional support, was essential to survival in addition to oncological therapy.

Except for ALP and albumin status, another variable included in the formula was leukocyte on behalf of the systemic inflammation associated with high mortality risk, which was in keeping with previous studies (7). In the setting of bone metastatic lung cancer, systemic inflammation was mainly due to increased tumor-induced bone resorption through the activation of a vicious circle between bone and metastases (24, 25). Tumor cells in the bone disrupt normal bone physiology, resulting in the release of inflammatory cytokines and growth factors, such as interleukin-1, interleurkin-6, fibroblast growth factor, platelet-derived growth factor, transforming growth factor-β, and so on from the bone matrix, which increases tumor cell growth in turn and promotes further bone disruption. This cyclic relationship increases metastatic lesions in the bone and eventually leads to numerous comorbidities including bone fracture, hypercalcemia, and systemic inflammation (23, 25).

Various demographic and clinical variables were reported that show inferior prognosis for LUAD with bone metastasis to include: male gender (21), smoking history (20), cachexia (7), malnutrition (7), poor physical status (21, 26), multiple and/or wearing bone metastases (4, 7, 26), *KRAS* mutation (7, 22), and without targeted therapy mutations (6, 8). In Meng’s study, which explored the factors affecting the survival of NSCLC patients with BOM, *EGFR* sensitive/*ALK* mutations, smoking and loss of weight were good, poor, and bad factors, respectively (6). In our study, which only included the LUAD with one to five BOM, loss of weight and without sensitive targeted-therapy mutations still were inferior prognostic factors; meanwhile, advanced T and N stage were also significantly associated with worsened survival.

The optimal treatment strategy should vary according to patients’ estimated survival time in the real word (27–29). A variety of scoring systems and predictive models had been established and verified for this purpose. For example, Pruksakorn et al. (21) developed a scoring system using gender and Eastern Cooperative Oncology Group score, which were significant prognostic factors, to estimate the survival time of lung cancer patients with bone metastasis. Meng et al. (6) proposed a graded prognostic assessment model for NSCLC patients with BOM that relied on smoking, *EGFR* sensitive/*ALK* mutations status, loss of weight, hypoalbuminemia, and primary site treated by surgery or radiotherapy. However, these scoring systems included metastasis of other sites, or many pathological types of lung cancer, or both. So, we recognized a need for a survival prediction model that is specific for LUAD patients with BOM. In addition, oncologists and radiologists were more likely to pay attention to advanced patients with oligometastatic disease, who would benefit from local treatment. So, in this study, we established a nomogram model to predict the 3- and 4-year survival rate of these patients, and the addition of hematological data indeed improved the predictive accuracy and clinical utility.

## Limitations

There are several limitations to this analysis. Most importantly, due to its retrospective nature, the bone metastatic status was assessed by non-homogeneous imaging techniques which had the different diagnosis capacity. Secondly, we were lacking in some data, such as C-reactive protein, cachexia, sarcopenia, and *KRAS* mutation status, which were essential to survival. Thirdly, treatments were also inconsistent, which may influence survival. Finally, the number of patients was limited, and further multi-center studies are needed to confirm this model.

## Conclusions

The survival time of LUAD patients with one to five BOM at initial diagnosis was significantly influenced by bone metabolism; nutritional and inflammatory indexes; loss of weight; EGFR-sensitive/ALK mutations; and T and N stage. A nomogram model based on hematological markers was developed in this study to guide physicians when estimating survival time for these patients.

## Data Availability Statement

The raw data supporting the conclusions of this article will be made available by the authors, without undue reservation.

## Ethics Statement

Ethical review and approval was not required for the study of human participants in accordance with the local legislation and institutional requirements. Written informed consent from the patients/participants OR patients/participants legal guardian/next of kin was not required to participate in this study in accordance with the national legislation and the institutional requirements.

## Author Contributions

Each author’s contribution for this manuscript is as follows: CM: Conceptualization, Methodology, Formal analysis, Investigation, Writing - Original Draft. FW: Conceptualization, Methodology, Formal analysis, Investigation. MC and HS: Investigation, Methodology. LZ and PW: Writing - Review and Editing. All authors contributed to the article and approved the submitted version.

## Funding

This research was found by Chinese National Key Research and Development Project (Grant No. 2018YFC1315601).

## Conflict of Interest

The authors declare that the research was conducted in the absence of any commercial or financial relationships that could be construed as a potential conflict of interest.

## Publisher’s Note

All claims expressed in this article are solely those of the authors and do not necessarily represent those of their affiliated organizations, or those of the publisher, the editors and the reviewers. Any product that may be evaluated in this article, or claim that may be made by its manufacturer, is not guaranteed or endorsed by the publisher.

## Abbreviations

LUAD, lung adenocarcinoma; NSCLC, non-small cell lung cancer; OS, overall survival; BOM, bone only metastasis; TNM, tumor-node-metastasis; SM, sensitive mutations; EGFR, epidermal growth factor receptor; ALK, anaplastic lymphoma kinase; TKIs, tyrosine kinase inhibitors; PLR, platelet to lymphocyte ratio; NLR, neutrophils to lymphocyte ratio; SII, systemic inflammatory index; ALP, alkaline phosphatase; LASSO, least absolute shrinkage and selection operator; PFS, progression free survival; C-index, concordance index; ROC, receiver operating characteristic; AUC, area under the curve; DCA, decision curve analysis.

## References

[1] Sung H Ferlay J Siegel RL Laversanne M Soerjomataram I Jemal A. Global Cancer Statistics 2020: GLOBOCAN Estimates of Incidence and Mortality Worldwide for 36 Cancers in 185 Countries. CA Cancer J Clin (2021) 71(3):209–49. doi: 10.3322/caac.21660 33538338

[2] Confavreux CB Girard N Pialat JB Bringuier PP Devouassoux-Shisheboran M Rousseau JC. Mutational Profiling of Bone Metastases From Lung Adenocarcinoma: Results of a Prospective Study (POUMOS-TEC). Bonekey Rep (2014) 3:580. doi: 10.1038/bonekey.2014.75 25328676 PMC4181073

[3] Ferlay J Steliarova-Foucher E Lortet-Tieulent J Rosso S Coebergh JWW Comber H. Cancer Incidence and Mortality Patterns in Europe: Estimates for 40 Countries in 2012. Eur J Cancer (2013) 49(6):1374–403. doi: 10.1016/j.ejca.2012.12.027 23485231

[4] Sugiura H Yamada K Sugiura T Hida T Mitsudomi T. Predictors of Survival in Patients With Bone Metastasis of Lung Cancer. Clin Orthop Relat Res (2008) 466(3):729–36. doi: 10.1007/s11999-007-0051-0 PMC2505203 18196360

[5] Cetin K Christiansen CF Jacobsen JB Norgaard M Sorensen HT. Bone Metastasis, Skeletal-Related Events, and Mortality in Lung Cancer Patients: A Danish Population-Based Cohort Study. Lung Cancer (2014) 86(2):247–54. doi: 10.1016/j.lungcan.2014.08.022 25240518

[6] Meng C Wei J Tian J Ma J Liu N Yuan Z. Estimating Survival and Clinical Outcome in Advanced non-Small Cell Lung Cancer With Bone-Only Metastasis Using Molecular Markers. J Bone Oncol (2021) 31:100394. doi: 10.1016/j.jbo.2021.100394 34703756 PMC8524192

[7] Chambard L Girard N Ollier E Rousseau JC Duboeuf F Carlier MC. Bone, Muscle, and Metabolic Parameters Predict Survival in Patients With Synchronous Bone Metastases From Lung Cancers. Bone (2018) 108:202–9. doi: 10.1016/j.bone.2018.01.004 29337225

[8] Lagana M Gurizzan C Roca E Cortinovis D Signorelli D Pagani F. High Prevalence and Early Occurrence of Skeletal Complications in EGFR Mutated NSCLC Patients With Bone Metastases. Front Oncol (2020) 10:588862. doi: 10.3389/fonc.2020.588862 33282740 PMC7689017

[9] Hellman S Weichselbaum RR. Oligometastases. J Clin Oncol (1995) 13(1):8–10. doi: 10.1200/JCO.1995.13.1.8 7799047

[10] Xu Q Zhou F Liu H Jiang T Li X Xu Y. Consolidative Local Ablative Therapy Improves the Survival of Patients With Synchronous Oligometastatic NSCLC Harboring EGFR Activating Mutation Treated With First-Line EGFR-TKIs. J Thorac Oncol (2018) 13(9):1383–92. doi: 10.1016/j.jtho.2018.05.019 29852232

[11] Iyengar P Wardak Z Gerber DE Tumati V Ahn C Hughes RS. Consolidative Radiotherapy for Limited Metastatic Non-Small-Cell Lung Cancer: A Phase 2 Randomized Clinical Trial. JAMA Oncol (2018) 4(1):e173501. doi: 10.1001/jamaoncol.2017.3501 28973074 PMC5833648

[12] Gomez D Tang C Zhang J Blumenschein G Hernandez M Lee J. Local Consolidative Therapy Vs. Maintenance Therapy or Observation for Patients With Oligometastatic Non-Small-Cell Lung Cancer: Long-Term Results of a Multi-Institutional, Phase II, Randomized Study. J Clin Oncol (2019) 37(18):1558–65. doi: 10.1200/JCO.19.00201 PMC6599408 31067138

[13] Palma DA Olson R Harrow S Gaede S Louie AV Haasbeek C. Stereotactic Ablative Radiotherapy Versus Standard of Care Palliative Treatment in Patients With Oligometastatic Cancers (SABR-COMET): A Randomised, Phase 2, Open-Label Trial. Lancet (2019) 393(10185):2051–8. doi: 10.1016/S0140-6736(18)32487-5 30982687

[14] Lopez Guerra JL Gomez D Zhuang Y Hong DS Heymach JV Swisher SG. Prognostic Impact of Radiation Therapy to the Primary Tumor in Patients With non-Small Cell Lung Cancer and Oligometastasis at Diagnosis. Int J Radiat Oncol Biol Phys (2012) 84(1):e61–7. doi: 10.1016/j.ijrobp.2012.02.054 PMC3919541 22503522

[15] Ashworth AB Senan S Palma DA Riquet M Ahn YC Ricardi U. An Individual Patient Data Metaanalysis of Outcomes and Prognostic Factors After Treatment of Oligometastatic non-Small-Cell Lung Cancer. Clin Lung Cancer (2014) 15(5):346–55. doi: 10.1016/j.cllc.2014.04.003 24894943

[16] Collen C Christian N Schallier D Meysman M Duchateau M Storme G. Phase II Study of Stereotactic Body Radiotherapy to Primary Tumor and Metastatic Locations in Oligometastatic Nonsmall-Cell Lung Cancer Patients. Ann Oncol (2014) 25(10):1954–9. doi: 10.1093/annonc/mdu370 25114022

[17] Petty WJ Urbanic JJ Ahmed T Hughes R Levine B Rusthoven K. Long-Term Outcomes of a Phase 2 Trial of Chemotherapy With Consolidative Radiation Therapy for Oligometastatic Non-Small Cell Lung Cancer. Int J Radiat Oncol Biol Phys (2018) 102(3):527–35. doi: 10.1016/j.ijrobp.2018.06.400 PMC6743975 30003996

[18] Farooqi A Ludmir EB Mitchell KG Antonoff MB Tang C Lee P. Increased Biologically Effective Dose (BED) to the Primary Tumor is Associated With Improved Survival in Patients With Oligometastatic NSCLC. Radiother Oncol (2021) 163:114–8. doi: 10.1016/j.radonc.2021.08.005 34419505

[19] Chansky K Detterbeck FC Nicholson AG Rusch VW Vallieres E Groome P. The IASLC Lung Cancer Staging Project: External Validation of the Revision of the TNM Stage Groupings in the Eighth Edition of the TNM Classification of Lung Cancer. J Thorac Oncol (2017) 12(7):1109–21. doi: 10.1016/j.jtho.2017.04.011 28461257

[20] Guo X Ma W Wu H Xu Y Wang D Zhang S. Synchronous Bone Metastasis in Lung Cancer: Retrospective Study of a Single Center of 15,716 Patients From Tianjin, China. BMC Cancer (2021) 21(1):613. doi: 10.1186/s12885-021-08379-2 34039303 PMC8152068

[21] Pruksakorn D Phanphaisarn A Settakorn J Arpornchayanon U Tantraworasin A Chaiyawat P. Prognostic Score for Life Expectancy Evaluation of Lung Cancer Patients After Bone Metastasis. J Bone Oncol (2018) 10:1–5. doi: 10.1016/j.jbo.2017.10.001 29321965 PMC5726457

[22] Lohinai Z Klikovits T Moldvay J Ostoros G Raso E Timar J. KRAS-Mutation Incidence and Prognostic Value are Metastatic Site-Specific in Lung Adenocarcinoma: Poor Prognosis in Patients With KRAS Mutation and Bone Metastasis. Sci Rep (2017) 7:39721. doi: 10.1038/srep39721 28051122 PMC5209707

[23] Stewart AF. Clinical Practice. Hypercalcemia Associated With Cancer. N Engl J Med (2005) 352(4):373–9. doi: 10.1056/NEJMcp042806 15673803

[24] Mundy GR. Metastasis to Bone: Causes, Consequences and Therapeutic Opportunities. Nat Rev Cancer (2002) 2(8):584–93. doi: 10.1038/nrc867 12154351

[25] Fornetti J Welm AL Stewart SA. Understanding the Bone in Cancer Metastasis. J Bone Miner Res (2018) 33(12):2099–113. doi: 10.1002/jbmr.3618 30476357

[26] Bae HM Lee SH Kim TM Kim DW Yang SC Wu HG. Prognostic Factors for non-Small Cell Lung Cancer With Bone Metastasis at the Time of Diagnosis. Lung Cancer (2012) 77(3):572–7. doi: 10.1016/j.lungcan.2012.05.094 22672969

[27] Lutz S Berk L Chang E Chow E Hahn C Hoskin P. Palliative Radiotherapy for Bone Metastases: An ASTRO Evidence-Based Guideline. Int J Radiat Oncol Biol Phys (2011) 79(4):965–76. doi: 10.1016/j.ijrobp.2010.11.026 21277118

[28] Patchell RA Tibbs PA Regine WF Payne R Saris S Kryscio RJ. Direct Decompressive Surgical Resection in the Treatment of Spinal Cord Compression Caused by Metastatic Cancer: A Randomised Trial. Lancet (2005) 366(9486):643–8. doi: 10.1016/S0140-6736(05)66954-1 16112300

[29] Pin Y Paix A Le Fèvre C Antoni D Blondet C Noël G. A Systematic Review of Palliative Bone Radiotherapy Based on Pain Relief and Retreatment Rates. Crit Rev Oncol/Hematol (2018) 123:132–7. doi: 10.1016/j.critrevonc.2018.01.006 29482774

